# Use of GLP-1 receptor agonist and risk of osteoporosis among patients with type 2 diabetes: a real-world study

**DOI:** 10.3389/fendo.2025.1586589

**Published:** 2025-05-21

**Authors:** Ming Chen, Yiming Lyu, Jingwei Zhao, Xianwei Han, Tao Huang, Tongtao Yang, Yong Zhou

**Affiliations:** ^1^ Department of Orthopedics, Tangdu Hospital, Airforce Medical University (Fourth Military Medical University), Xi’an, Shanxi, China; ^2^ Medmotion Clinic, Shanghai, China

**Keywords:** type 2 diabetes, osteoporosis, GLP-1 receptor agonists, electronic medical records, cohort study, bone metabolism, fracture risk

## Abstract

**Background:**

Type 2 diabetes mellitus (T2DM) is an independent risk factor for osteoporosis, increasing the risk of fractures and poor prognosis. Recent studies suggest that glucagon-like peptide-1 receptor agonists (GLP-1 RAs) may play a protective role in bone metabolism. However, limited evidence exists on their effect on osteoporosis incidence in T2DM patients. This study aimed to evaluate the association between GLP-1 RA use and osteoporosis risk in a real-world cohort of elderly T2DM patients.

**Methods:**

This retrospective cohort study utilized electronic medical records (EMRs) from Tangdu Hospital, Xi’an, China, between January 1, 2012, and December 31, 2023. Patients with T2DM who had at least two clinical visits annually and no prior osteoporosis diagnosis (ICD-10: M80-M82) at baseline were included. The primary outcome was the incidence of osteoporosis during follow-up. Cox proportional hazards models were used to evaluate the association between GLP-1 RA use and osteoporosis risk, adjusting for age, sex, BMI, blood pressure, lipid profile, renal function, osteocalcin, vitamin D levels, HbA1c, statin use, antihypertensive medication use, and smoking status. Subgroup analyses were conducted to assess potential effect modifications.

**Results:**

A total of 1,845 patients with T2DM were included, of whom 676 (36.6%) developed osteoporosis during follow-up. Among the 256 patients who received GLP-1 RAs, the incidence of osteoporosis was significantly lower than in those who did not receive GLP-1 RAs (P < 0.01). In the fully adjusted Cox model, GLP-1 RA use was associated with a significantly reduced risk of osteoporosis compared to non-users (hazard ratio [HR] = 0.69, 95% confidence interval [CI] =0.45-0.84, P < 0.05). Subgroup analyses indicated that the protective effect of GLP-1 RAs was consistent across age, sex, BMI, smoking status, and antihypertensive medication use (P for interaction > 0.05).

**Conclusion:**

In patients with T2DM, patients with treatment of GLP-1 RAs resulted in lower risks of osteoporosis than those without treatment of GLP-1 RAs. These findings support the potential bone-protective effects of GLP-1 RAs but further randomized controlled trials (RCTs) or large-scale database analyses are needed to confirm these observations and guide clinical recommendations.

## Introduction

With the change of lifestyle and diet structure, the prevalence of type 2 diabetes (T2DM) is on the rise worldwide, and associated complications pose a threat to the life span and quality of life of patients. In 2015, there were about 415 million patients with diabetes in the world, which is expected to increase to 642 million by 2040 (1, 2)

Osteoporosis is a systemic bone disease characterized by a decrease in bone density, a reduction in bone mass, and an increase in bone fragility due to various reasons. The primary risk of osteoporosis is fractures, especially for elderly osteoporosis where the mortality rate from fractures is extremely high, resulting in significant expenditure of public healthcare resources worldwide (3, 4). Studies show that diabetes is an independent risk factor for osteoporosis. The incidence rate of osteoporosis in elderly T2DM patients is significantly higher than that of the general population, with a poor prognosis (5, 6).

Glucagon-like peptide-1 (GLP-1) is one of the incretin hormones which are defined as intestinal hormones released in response to nutrient ingestion, which potentiate the glucose-induced insulin response. GLP-1 receptor agonist (GLP-1 RAs) are mostly approved for T2DM in China till 2023, including but not limited to Liraglutide, Semaglutide and Dulaglutide. In recent years, clinical studies found that GLP-1 receptor agonist (GLP-1 RAs) and its analogues can significantly improve bone metabolism in diabetes patients, promoting osteoblastic differentiation, and play an anti-diabetic osteoporosis role (7). Animal experiments have further proved that GLP-1 RAs and its analogues presents a good anti-osteoporosis effect on postmenopausal osteoporosis, glucocorticoid osteoporosis, and senile osteoporosis (8, 9).

However, there are few studies on the impact of GLP-1 RAs on the incidence rate of osteoporosis. Therefore, this study aims to explore the impact of GLP-1 RAs on the occurrence of osteoporosis in elderly T2DM patients through a real-world cohort study.

## Method

### Study population

Tangdu Hospital, located in Xi’an, Shaanxi Province, China, is a leading tertiary-level medical institution renowned for its advanced clinical services, medical research, and education. The hospital is affiliated with the Air Force Medical University (Fourth Military Medical University, FMMU) and operates as a key medical center serving both military personnel and the general public. We extracted data from the electronic medical records (EMRs) from Tangdu Hospital since 2012. Patients with type 2 diabetes, who were on regular visits with at least two clinical visits annually at Tangdu Hospital were identified for this study. We obtained written approval from the Institutional Review Boards of Tangdu Hospital (K202409-15). We did not obtain informed consent from individual participants involved in our study because we used anonymized data compiled from EMRs.

**Figure 1:**
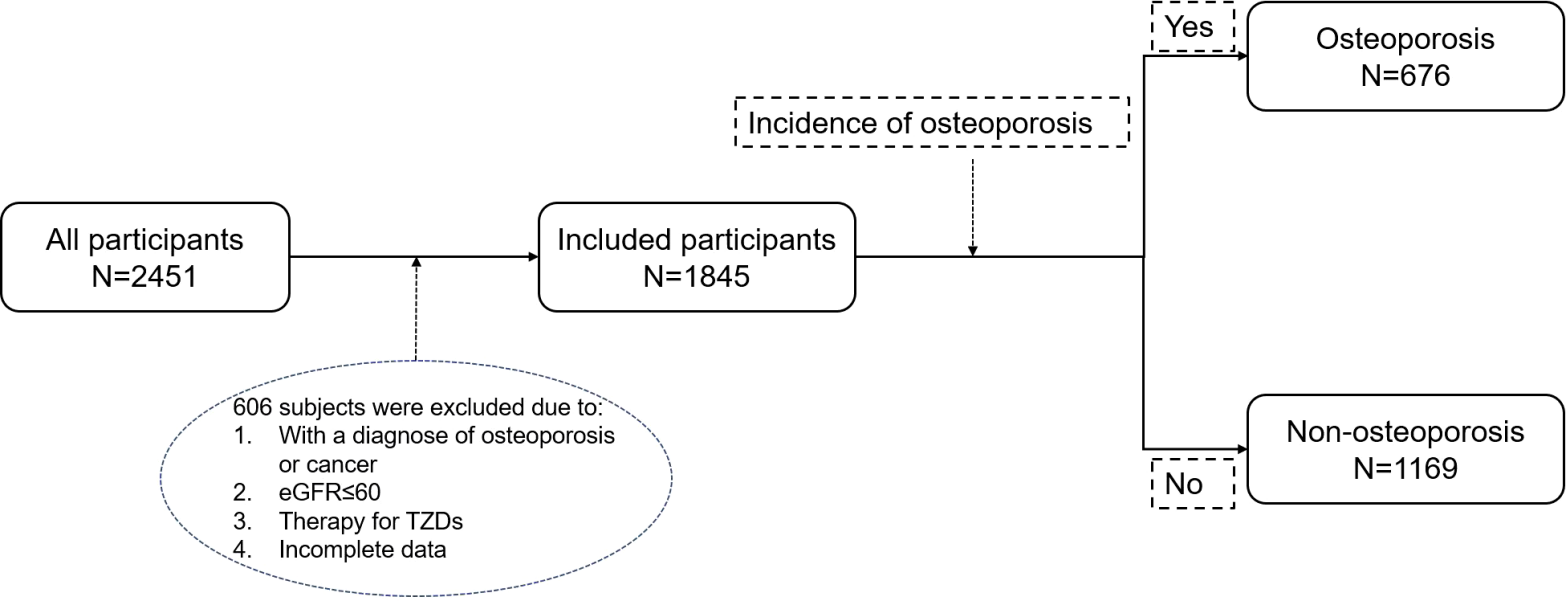
Flow chart of the study.

From January 1, 2012, to December 31, 2023, a total of 2,451 patients with type 2 diabetes were enrolled at Tangdu Hospital, of which 606 patients were excluded due to the following reasons: 1 When undergoing screening, the diagnosis of osteoporosis or any malignant tumor has been confirmed. 2. eGFR ≤ 60 mL/min/1.73 m². 3. Treatment with TZDs. 4. Relevant data is missing. Ultimately, 1,845 patients were included in the analysis and were divided into two groups based on whether they developed osteoporosis during the follow-up period: the osteoporosis group (n=676) and the non-osteoporosis group (n=1169) (**Figure 1**).

### Data collection and covariates

Demographic data collected included age, sex, and body mass index (BMI), which were recorded at baseline. Clinical parameters such as systolic blood pressure (SBP) and diastolic blood pressure (DBP) were also extracted from EMRs. Laboratory measures included low-density lipoprotein cholesterol (LDL-c), triglycerides (TG), high-density lipoprotein cholesterol (HDL-c), total cholesterol (TC), serum creatinine (Scr), estimated glomerular filtration rate (eGFR), osteocalcin (OCN), vitamin D (VitD), and hemoglobin A1c (HbA1c), all of which were obtained from routine clinical testing.

Medication usage was assessed using prescription records. Data on the use of GLP-1 RAs, statins, and antihypertensive medications were extracted. The study also recorded lifestyle factors, such as current smoking status, based on patient self-reports documented in the medical histories within the EMRs dataset.

### Baseline determination, follow-up and primary outcome

Baseline was defined as the date of the first recorded clinical encounter for T2D in the EMRs, provided that no prior diagnosis of osteoporosis was present at that time. Patients with a documented osteoporosis diagnosis before or on the baseline date were excluded from the study. The primary outcome of this study is the incidence of osteoporosis. To evaluate osteoporosis outcomes, baseline bone health markers and longitudinal incidence of osteoporosis were tracked through follow-up records in the EMR system. The diagnosis of osteoporosis was identified using International Classification of Diseases, 10th Revision (ICD-10) codes recorded in the EMRs. Patients were classified as having osteoporosis if they had at least one documented diagnosis code for osteoporosis (ICD-10: M80-M82) in their medical history. The follow-up period for each participant began from the baseline date and continued until the earliest occurrence of one of the following events: a new osteoporosis diagnosis, loss to follow-up, death, or the end of the study period. Person-years of follow-up were calculated for each participant as the time from baseline to the date of osteoporosis diagnosis, censoring event (loss to follow-up or death), or study end date, whichever occurred first. The total person-years were determined by summing up the follow-up durations of all participants.

### Statistical analysis

Baseline characteristics of participants were summarized as mean ± standard deviation (SD) for continuous variables and percentages for categorical variables. Differences between groups (osteoporosis vs. non-osteoporosis) were compared using independent t-tests for continuous variables and chi-square tests for categorical variables. The primary analysis examined the association between GLP-1 RAs use and osteoporosis incidence using Cox proportional hazards regression models. Three models were constructed: Model 1 (crude model), Model 2 (adjusted for age and sex), and Model 3 (fully adjusted for potential confounders including BMI, blood pressure, lipid profile, renal function, osteocalcin (OCN), vitamin D levels, HbA1c, statin use, antihypertensive medication use, and smoking status). Hazard ratios (HRs) and 95% confidence intervals (CIs) were reported. To assess the impact of follow-up duration, Cox models were stratified by ≤6 months, ≤12 months, and ≤24 months. Additionally, subgroup analyses were conducted to evaluate potential effect modification by sex, age, BMI, smoking status, and antihypertensive medication use, with P-values for interaction calculated to determine statistical significance. The proportional hazards assumption was tested using Schoenfeld residuals, and model fit was assessed using likelihood ratio tests. All statistical analyses were performed using R software (version 4.3.3), with a significance level of P < 0.05 for two-sided tests.

## Results

baseline characteristics and concomitant medication were shown in **Table 1**. The average age of patients in osteoporosis group (63.05 ± 11.53, year) was younger than those in Non-osteoporosis group (66.67 ± 11.43, year). Thosw with GLP-1 therapy had a lower average BMI (22.85 ± 7.29 vs 23.97 ± 7.86, kg/m^2^), and a higher proportion of males (52.02%, 36.27%). Baseline blood pressure, blood lipids (TG, TC, LDL-c, HDL-c), serum creatinine, serum OCN and Vit D had no difference between 2 groups. A total of 120 patients (17.99%) with osteoporosis and 136 patients(11.63%)without osteoporosis were treated with an GLP-1 RAs, respectively. 56.37% in the osteoporosis group and 64.67% in the non-osteoporosis group were treated with Statins, and 67.17% in the osteoporosis group and 80.07% in the non-osteoporosis group were using anti-hypertensive medications.

**Table 1:** Baseline characteristics of participants based on incidence of osteoporosis.

Characteristic	Osteoporosis (n=676)	Non-osteoporosis (n=1169)
Age (years)	63.05 ± 11.53	66.67 ± 11.43*
BMI (kg/m^2^)	22.85 ± 7.29	23.97 ± 7.86*
Male (%)	347 (52.02%)	424 (36.27%)*
Systolic blood pressure (mmHg)	132.37 ± 12.33	132.24 ± 10.43
Diastolic blood pressure (mmHg)	77.38 ± 7.90	75.56 ± 6.69
LDL-c (mmol/L)	2.65 ± 0.84	2.59 ± 0.77
TG (mmol/L)	1.14 ± 0.29	1.12 ± 0.32
HDL-c (mmol/L)	1.36 ± 0.22	1.36 ± 0.23
TC (mmol/L)	4.53 ± 1.00	4.50 ± 0.91
Scr (mg/dL)	0.99 ± 0.17	0.98 ± 0.17
eGFR	96.90 ± 19.89	96.52 ± 19.79
OCN (ug/L)	18.03 ± 2.82	17.50 ± 2.45
VitD (IU)	15.95 ± 3.56	16.55 ± 2.91
HbA1c (%)	7.50 ± 1.73	7.46 ± 1.41
GLP-1 use (%)	120 (17.99%)	136 (11.63%)*
Statin use (%)	376 (56.37%)	756 (64.67%)*
Anti-hypertension medications (%)	448 (67.17%)	936 (80.07%)*
Current smoking (%)	63 (9.45%)	97 (8.30%)

*P<0.05.

**Figure 2:**
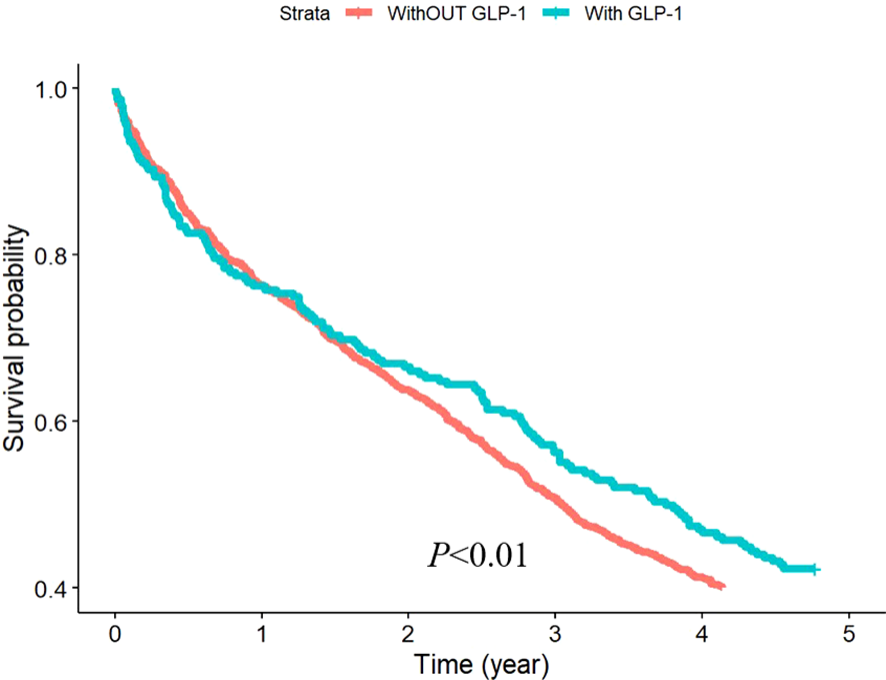
KM curve for osteoporosis.

To evaluate the effect of GLP-1 RAs on osteoporosis, we divided the patients into two groups based on whether they were treated with GLP-1 RAs or not. Among 1845 patients, 256 were treated with GLP-1, accounting for 13.88% of the total number of patients. The incidence of osteoporosis was significantly lower in the GLP-1 RAs treatment group than in the other group (**Figure 2**).

To assess the impact of follow-up duration, Cox models were stratified by ≤6 months, ≤12 months, and ≤24 months. Only in the short duration (<6 months) of follow-up, GLP-1 therapy had no significant relation to osteoporosis in fully-adjusted model (**Table 2**). Subgroup analyses were conducted to evaluate potential effect modification by sex, age, BMI, smoking status, and antihypertensive medication use. Test for interaction indicated that the effects of GLP-1 RAs on osteoporosis was not significantly affected by gender, age, BMI, smoking, and antihypertensive drugs (all P>0.05, **Table 3**).

**Table 2:** Main effect.

	No. of cases	Person-years	Non-GLP-1 users	GLP-1 users	95%CI
Full follow up	1845	5327.5			
Model 1			1.00	0.58	0.41-0.78
Model 2			1.00	0.64	0.48-0.83
Model 3			1.00	0.69	0.45-0.84
≤6	284	144.6			
Model 1			1.00	0.68	0.49-0.99
Model 2			1.00	0.89	0.65-1.02
Model 3			1.00	0.98	0.80-1.26
≤12	434	373.2			
Model 1			1.00	0.66	0.40-0.81
Model 2			1.00	0.74	0.47-0.89
Model 3			1.00	0.82	0.61-0.98
≤24	659	1057.7			
Model 1			1.00	0.60	0.42-0.83
Model 2			1.00	0.70	0.53-0.89
Model 3			1.00	0.79	0.55-0.92

Model 1: crude model.

Model 2: adjusted for age and sex.

Model 3: adjusted for other variables including BMI, blood pressure, lipid profile, renal function, osteocalcin, vitamin D levels, HbA1c, statin use, antihypertensive medication use, and smoking status.

**Table 3:** Subgroup for cox regression of GLP-1 therapy and osteoporosis.

Subgroup	No. of cases	Person-years	HR	lower	upper	P for interaction
Sex	1845	5327.5				0.37
female	1074	2806.	0.75	0.64	0.89	
male	771	2520.6	0.57	0.52	0.62	
Age						0.354
age>75	357	832.4	0.96	0.73	1.21	
age<=75	1488	4495.1	0.5	0.43	0.57	
BMI						0.264
BMI>=24	761	2105.6	0.86	0.79	0.94	
BMI<24	1084	3221.9	0.71	0.65	0.76	
Current smoking						0.941
No	1685	4833.9	0.17	0.36	0.82	
Yes	160	493.6	0.78	0.62	0.99	
Anti-hypertension medications						0.187
No	461	1554.6	0.72	0.62	0.83	
Yes	1384	3772.9	0.74	0.69	0.79	

## Discussion

In the present study, patients with type 2 diabetes who received GLP-1 RAs had a lower risk of osteoporosis. The results of subgroup analysis showed that the interaction analysis of risk factors related to osteoporosis included gender, age, BMI, smoking and the use of antihypertensive drugs do not significantly affect the protective effect of GLP-1 RAs on osteoporosis.

T2DM patients have low bone mineral density and increased fracture risk, especially hip and vertebral fractures (10–12). Meta-analysis illustrated that the pooled prognosis rate of osteoporosis among T2DM patients in mainland China was 37.8% (13). At the cellular and molecular levels, the characteristic of T2DM is reduced bone turnover, indicating changes in bone cell behavior (14). In addition, low testosterone and vitamin D levels and high plasma osteocalcin are common characteristics of type 2 diabetes patients (14). Anti diabetes drugs can affect bone metabolism, for example, TZDs are selective agonists of PPAR - γ family nuclear receptors. Meta analysis shows that TZDs treatment significantly reduces bone density in the forearm, lumbar spine, and total hip joints, and this effect is irreversible even after stopping TZDs for one year (15). TZDs treatment can significantly increase the risk of fractures in women, while it has no effect in men (16).

GLP-1 is produced by the post-translational processing of the glucagon gene in intestinal endocrine cells (17). GLP-1 receptor is expressed in several regions of the endocrine pancreas, gastrointestinal tract, lungs, heart, kidneys, and brain (18), and GLP-1 RAs is a half-life prolonging GLP-1 with stronger resistance to the degradation of dipeptidyl peptidase-4 (DPP-4). GLP-1 RAs reduce the risk of fractures in T2DM patients, and its beneficial effects are related to the duration of treatment. Compared with placebo and other anti-diabetes drugs, Liraglutide can significantly reduce the risk of fracture in patients with type 2 diabetes (19). GLP-1 receptor agonist (GLP-1 RAs) are mostly approved for T2DM in China till 2023, including but not limited to Liraglutide, Semaglutide and Dulaglutide. However, it’s a pity in our study that we did not collect the commercial brand to perform subgroup analysis for different types of GLP-1 RAs.

Our study also demonstrated that GLP-RAs could significantly reduce the risk of osteoporosis in T2DM patients, consistent with the find with Liraglutide. Furthermore, we found that even in one-year follow up, GLP-1 can reduce the risk of osteoporosis and the HR is lower with the longer duration of follow up, which means GLP-1 RAs might have a lasting influence on bone metabolism. Subgroup analysis showed that GLP-1 could reduce the risk of osteoporosis in both man and female diabetic patients. Although the HR for male was numerically lower for female (0.57 vs 0.75), p for interaction was 0.37 which means there was no difference between male and female. The possibility that GLP-1RAs could improve bone metabolism, which is essential for skeletal health, is of major interest and suggests that GLP-1 RAs could benefit the rising number of elderly T2DM patients with osteoporosis and high fracture risk.

Our study showed no differences for VitD and OCN at baseline between those with osteoporosis and without osteoporosis at follow-up. Whether VitD or OCN could predict osteoporosis remained uncertain and this resulted in a non- significant difference in the levels of OCN and Vit D at baseline in our study. Hypertension has been increasingly recognized as a potential risk factor for reduced bone mineral density and osteoporotic fractures, potentially through shared pathophysiological pathways such as chronic inflammation, oxidative stress, and impaired calcium metabolism. Although our study did not detect the difference at baseline between osteoporosis and non- osteoporosis, this may result from the large proportion of anti-hypertension therapy.

It can be easy to reach out that weight loss by GLP-RAs can have benefits on bone. Additionally, GLP-1 may exert anti-osteoporosis effects through different mechanisms. Liraglutide was found to activate osteoblast proliferation by promoting the Wnt signaling pathway and p-AMPK/PGC1 α signaling pathway, and inhibit osteoclast activation by suppressing the OPG/RANKL/RANK signaling pathway through anti-inflammatory, antioxidant, and anti autophagic pathways, thereby reversing bone loss (20). Also, GLP-1 RAs mainly improves bone microstructure and bone strength by reducing bone resorption and promoting bone formation and reverses glucocorticoid induced osteoporosis (9). In addition, GLP-1 RAs, to some extent, promotes osteoblast production and inhibits bone resorption in obese type 2 diabetes rats, which may be partially mediated by AGEs/AGE/ROS pathway (21).

There are certain limitations of this study that should be noted. First, the study population is limited to the one center with Chinese population, which may introduce bias in the source of subjects, and the conclusions should be cautiously interpretated. Secondly, we did not collect the parameters like BMD or RANKL/OPG, physical activities and diets, glucose parameters at follow-up which may have potential relationship to osteoporosis, which may lower the power of study. Moreover, the commercial brand and the duration of GLP-1 were not collected in our study, leading to bias in our study. Furthermore, due to the retrospective design and limitations in our study, RCTs or database analysis in a larger number of patients may be the next step to get more robust conclusions.

## Conclusion

In patients with T2DM, patients with treatment of GLP-1 RAs resulted in lower risks of osteoporosis than those without treatment of GLP-1 RAs. Physicians should raise more awareness of the relationship between GLP-1 RAs and osteoporosis in clinical practice. Further randomized controlled trials (RCTs) or large-scale database analyses are needed to confirm these observations and guide clinical recommendations.

## Data Availability

The raw data supporting the conclusions of this article will be made available by the authors, without undue reservation.
